# PICCOS: study protocol for pressurised intraperitoneal aerosolised chemotherapy (PIPAC) in the management of cancers of the colon, ovary and stomach: a randomised controlled, phase II trial of efficacy in peritoneal metastases

**DOI:** 10.1515/pp-2025-0023

**Published:** 2026-05-01

**Authors:** Sadie E.F. Jones, Elena Brogden, Jamie Murphy, Christopher J. Peters, Jonathan Frost, Richard Adams, Sarah Gwynne, Emma Hudson, Kitrick Perry, Angela Casbard, Rebecca Hamilton, Lisette Nixon, Philip Markham, Huda Mohamed, Gina Brown, Jane Blazeby, Deborah Fitzsimmons, Anuoluwa Ajakaiye, Leona M. Batten, Maureen Edgar, Amy Case, Peter Kyle, Sophie Tate, Karen Arndell, Daisy Elliott, Rhiannon Macefield, David Chuter, Harry Hall, Joy Garfitt, Jared Torkington

**Affiliations:** Cardiff and Vale University Health Board, University Hospital of Wales, Cardiff, UK; Centre for Trials Research, Cardiff University, Cardiff, UK; Department of Surgery and Cancer, Imperial College London, London, UK; Royal United Hospitals Bath NHS Foundation Trust, Bath, UK; Swansea Bay University Health Board, Southwest Wales Cancer Centre, Swansea, UK; Velindre University NHS Trust, Velindre Cancer Hospital, Cardiff, UK; Imperial College Healthcare NHS Trust, London, UK; National Institute for Health Research Bristol Biomedical Research Centre, University Hospitals Bristol and Weston NHS Foundation and University of Bristol, Bristol, UK; Swansea Centre for Health Economics, Swansea University, Swansea, UK; Health Technology Wales, NHS Wales, Cardiff, UK; Independent Cancer Patients Voice, London, UK; Brecon, Wales; London, England

**Keywords:** peritoneal metastases, randomised controlled trial, PIPAC, response evaluation criteria in solid tumours (RECIST), quality of life

## Abstract

**Objectives:**

Peritoneal metastases (PM) represent a significant unmet need requiring urgent development of treatment options to improve patient prognosis and quality of life (QoL). A novel anti-cancer technology is Pressurised IntraPeritoneal Aerosolised Chemotherapy (PIPAC). PIPAC aims to improve target specificity of anti-cancer therapies by delivering medication as an aerosol directly to the peritoneum at laparoscopy. Early phase I/II studies suggest that PIPAC has the potential to improve treatment efficacy and cancer outcomes whilst maintaining or improving QoL through less systemic drug absorption. PICCOS aims to determine whether PIPAC can improve peritoneal progression free survival (pPFS) in patients with PM from, compared to SACT alone.

**Methods:**

PICCOS is a phase II, multi-centre, superiority, randomised controlled trial (ISRCTN 17575409). It aims to determine whether PIPAC (given alone or in combination with systemic anti-cancer therapy (SACT)) can improve peritoneal progression free survival (pPFS) as per RECIST (V1.1) in patients with PM, compared to SACT alone. Key secondary outcome measures include quality of life, safety, overall survival and progression free survival. 216 patients with non-resectable PM from colorectal cancer, platinum resistant ovarian cancer and gastric cancer will be recruited. Patients will be randomised to receive either standard of care SACT alone or PIPAC in combination with (colorectal, gastric groups) or without (ovarian group) standard of care SACT. Median pPFS will be estimated using the Kaplan–Meier method. The log-rank test, stratified by prognostic factors, will be used to compare PFS distributions.

**Results:**

PIPAC is expected to achieve improved peritoneal progression free survival for patients in comparison to those treated with standard care.

**Conclusions:**

PICCOS aims to provide much needed, high-quality evidence on the efficacy of PIPAC in treating PM.

## Introduction

### Background and rationale

Colorectal, ovarian and gastric cancers account for 57,000 new cancer diagnoses a year in the UK and 24,500 deaths. When these tumours spread or recur within the peritoneum, they are generally deemed incurable as no effective treatment strategy exists [1], [2], [3]. As many as 10 % of colorectal, 50 % of ovarian and 14 % of gastric cancers will have peritoneal metastases (PM) at the time of presentation [4]. PM are not only associated with poor prognosis but also significant symptomatology. Patients are left with an uncertain future, reduced quality of life (QoL) and very few treatment options. It is widely acknowledged by the clinical community, that standard systemic anti-cancer therapy (SACT) across all three disease groups is not having the desired impact. Peritoneal metastatic disease therefore continues to be a significant and urgent, unmet clinical need.

Fortunately, the treatment of PM is evolving with the development of advanced therapies and novel techniques by which to deliver them. Intraperitoneal (IP) chemotherapy represents one such novel approach. IP chemotherapy has a growing body of evidence demonstrating clinical efficacy through the delivery of anti-cancer therapy directly to target, thereby maximising tumour penetration whilst minimising systemic toxicities [5]. IP chemotherapy can be delivered as a heated solution in the form of Hyperthermic IntraPeritoneal Chemotherapy (HIPEC) or as an aerosol in the form of Pressurised IntraPeritoneal Aerosolised Chemotherapy (PIPAC). The detailed indications for, and efficacy of HIPEC fall outside the scope of this protocol paper but typically it is delivered at the time of open cytoreductive surgery and is currently considered in patients with surgically resectable disease [6]. PIPAC involves introducing aerosolised chemotherapy into the abdomen under the pressure created by conventional laparoscopy [7]. Much of the existing clinical evidence for PIPAC is in patients with unresectable disease and therefore, in patients associated with a particularly poor prognosis [8], [9], [10], [11], [12], [13], [14], [15], [16], [17], [18]. For example, a median progression free survival (PFS) of 6 months and overall survival (OS) of 16.3 months is reported for patients with PM from unresectable colorectal cancer [19]; for patients with PM from gastric cancer, OS is reported at 3–6 months, extended to 9–11 months with standard SACT [20], 21]. For patients with PM from ovarian cancer, initial platinum based systemic chemotherapy regimens tend to have better efficacy. Currently, PIPAC has largely been explored in the context of platinum resistant disease in which median PFS of 3–4 months and OS of 9–13 months [22], [23], [24] are reported. Phase I and non-randomised phase II studies have indicated that PIPAC may be associated with improved survival and maintained or improved QoL. In colorectal cancer, PIPAC has been associated with OS figures ranging from 8 to 27 months [11], [25], [26], [27], 6–22 months in ovarian cancer [10], 17], [27], [28], [29] and 8–19 months in gastric cancer [11], [12], [13], [14], [15], [16, [30], [31], [32], [33], [34], [35]. These data however, come from studies with heterogenous design (varied study populations, treatment regimens) that make clinical interpretation difficult. The PICCOS trial (funded by the National Institute for Health Research, Efficacy and Mechanism Evaluation fund (NIHR EME)) aims to generate high-quality evidence, not only assessing the efficacy of PIPAC treatment of PM compared to standard of care (SOC), but also in evaluating QoL, in the form of a randomised controlled trial (RCT).

The evidence base for PIPAC has currently reached stage 2 (development and exploration) of the IDEAL framework [36], 37] with no current published RCTs. A core set of patient reported outcome measures for peritoneal surface malignancies has been developed although it has not yet been widely used [38]. The PICCOS trial is a phase II, RCT designed to provide high-quality evidence regarding the efficacy of PIPAC in improving PFS in patients with PM from colorectal, ovarian and gastric cancer. PICCOS will provide IDEAL framework stage 2b level evidence for PIPAC and provide direction for future phase III trials.

## Patients and methods

### Trial design

**Figure 1:**
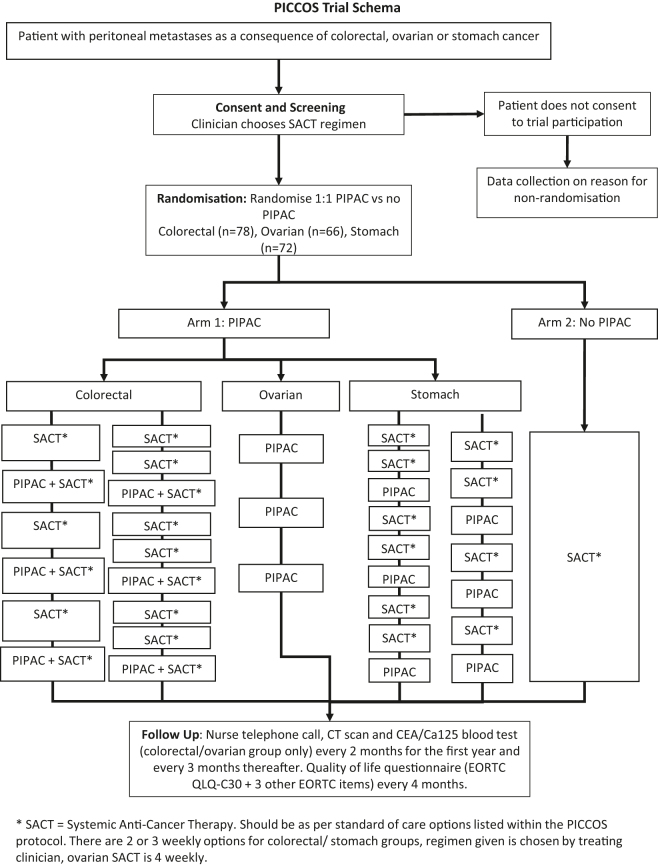
The PICCOS trial schema.

This is a phase II, multicentre, superiority, RCT. It has a basket design with a master protocol covering the overarching research methodology, and embedded individual cancer site specific protocols, sample sizes and analysis plans. Patients will be randomised in a 1:1 ratio to either SOC SACT or the PIPAC intervention arm (Figure 1).

### Objectives

The primary objective is to determine whether PIPAC given with (colorectal, gastric) or instead of (ovarian) SACT improves peritoneal progression free survival (pPFS) compared to SOC SACT.

#### Secondary objectives

– To determine how PIPAC impacts QoL compared to SOC.
– To determine the safety of PIPAC in terms of the proportion of patients experiencing toxicity and/or surgical complications, compared to the SACT only groups.
– To determine the proportion of intervention arm patients who complete three PIPAC procedures.
– To evaluate OS.
– To evaluate overall PFS.
– To determine peritoneal specific Overall Response Rate (ORR) and Disease Control Rate (DCR).

#### Tertiary/exploratory objectives

– To determine the feasibility of randomisation.
– To evaluate and compare between the two arms the response of Carcinoembryonic Antigen (CEA) (in colorectal cancer) and Cancer Antigen 125 (CA125) (in ovarian cancer) with the radiological response of peritoneal disease.
– To cross correlate the radiological evaluation to assess scan effectiveness and mismatches with Peritoneal Cancer Index (PCI) scoring at laparoscopy.
– To explore the effect of further anti-cancer treatment on OS.

### Study setting

PICCOS will be carried out at participating NHS hospitals within the UK with the potential for international expansion. All sites (approximately n = 40) will recruit patients and deliver SACT. It is anticipated that approximately ten of the sites, geographically spread across the UK, will also deliver PIPAC treatment. The remaining sites will refer patients to PIPAC sites when randomised for PIPAC treatment. The trial is being conducted by the Centre for Trials Research (CTR), Cardiff University (CU) and sponsored by Cardiff and Vale University Health Board (CVUHB). A list of current open sites can be obtained by contacting PICCOS@cardiff.ac.uk.

### Eligibility criteria

Patients are eligible for the trial if they meet all the relevant inclusion criteria and none of the relevant exclusion criteria (Tables 1–4).

**Table 1:** Inclusion and exclusion criteria relevant to all disease groups.

All disease groups	
Inclusion criteria	Exclusion criteria
16 years and older	Any prior malignancy not considered in complete remission for at least 2 years, excluding non-melanoma skin cancer.
Visible (measurable or non-measurable) peritoneal lesion(s) on CT imaging as per RECIST v1.1	Pregnant or breastfeeding.
ECOG performance status 0–1	Bevacizumab/Aflibercept should not be used in either arm (minimum 4 weeks from any prior Bevacizumab/Aflibercept).
Adequate bone marrow, liver and kidney function (within 7 days prior to randomisation): Neutrophil ≥1.5 × 10^9^/L White blood cells ≥3.0 × 10^9^/L Platelets ≥100 × 10^9^/L Haemoglobin ≥90 g/L Serum bilirubin ≤3 × ULN ALT/AST ≤2.5 × ULN (if both done, both must meet criteria) Creatinine clearance ≥50 mls/min	Untreated central nervous system disease or symptomatic central nervous system metastasis, history or evidence of thrombotic or haemorrhagic disorders not considered currently in complete remission.
Fit enough to receive full dose of SACT in cycle 1 as defined in the protocol.	Contraindication to any drug contained in the chemotherapy regimen.
Ability to provide informed consent obtained prior to any trial-specific screening procedures.	Medical, geographical, sociological, psychological or legal conditions that would prevent the patient from completing the trial or signing the informed consent.
	Unresolved bowel obstruction or parenteral nutrition or gastric tube.
	Contraindication to surgery.
	Participating in other oncological trials that may impact on endpoint.
	Life expectancy <3 months.

CT, computed tomography; RECIST, response evaluation criteria in solid tumours; ECOG, eastern cooperative oncology group; ALT, alanine transaminase; AST, aspartate transaminase; ULN, upper limit of normal.

**Table 2:** Inclusion and exclusion criteria specific to colorectal cancer patients.

Colorectal group specific criteria	
Inclusion criteria	Exclusion criteria
PM from histologically proven primary adenocarcinoma of the colorectum	Extra-peritoneal metastases except for: 1 Retroperitoneal lymph nodes <2 cm 2 Lung metastases; less than 5 lung metastases of which none are >1 cm 3 Solid organ metastases deemed asymptomatic and non-progressive by MDT

	Prior systemic therapy for colorectal cancer in the last 6 months.
	Dihydropyrimidine dehydrogenase deficiency (DPYD) variant detected.
	Micro-satellite instability (MSI) high.
	Previous CRS or HIPEC.

**Table 3:** Inclusion and exclusion criteria specific to ovarian cancer patients.

Ovarian group specific criteria	
Inclusion criteria	Exclusion criteria
PM from histologically confirmed primary epithelial ovarian, tubal, or primary peritoneal platinum-resistant carcinoma (including clinical recurrence, refractory disease, or persistent disease within 6 months of last chemotherapy).	Extra-peritoneal metastases except for retroperitoneal lymph nodes
	Parenchymal liver or spleen metastases.
	Malignant pleural effusion.
	Non-epithelial pathology subtype.
	Peritoneal disease, amenable to surgical resection.

**Table 4:** Inclusion and exclusion criteria specific to stomach cancer patients.

Stomach group specific criteria	
Inclusion criteria	Exclusion criteria
PM from histologically proven primary adenocarcinoma (any subtype) of stomach or Siewert type 3 gastro-oesophageal junction tumour (any HER2 status or CPS).	Extra-peritoneal metastases except for retroperitoneal lymph nodes.
	Prior SACT, radiotherapy or surgery for gastric cancer.
	Gastric or duodenal stent *in situ*.
	Gastro-oesophageal junction Siewert type 1 or type 2 tumour.
	Symptoms and/or radiology suggestive of impending and/or current bowel obstruction.
	Uncontrolled and persistent ascites.
	MSI high.
	DPYD variant detected.
	Previous CRS or HIPEC.

### Interventions

Prior to randomisation, the treating investigator will select the SACT regimen most appropriate to the individual patient from the options available in the protocol (see [Sec j_pp-2025-0023_s_005]). Following randomisation, treatment should commence within 2 weeks for colorectal and gastric groups and within 2–3 weeks for the ovarian intervention arms. Participants who show extraperitoneal progression, in any arm, and have not yet completed the treatment period may switch to an alternative (colorectal and stomach) SACT regimen or commence a SACT regimen (ovarian) listed in the protocol at the discretion of their Principal Investigator (PI).

#### Colorectal intervention arm

**Figure 2:**
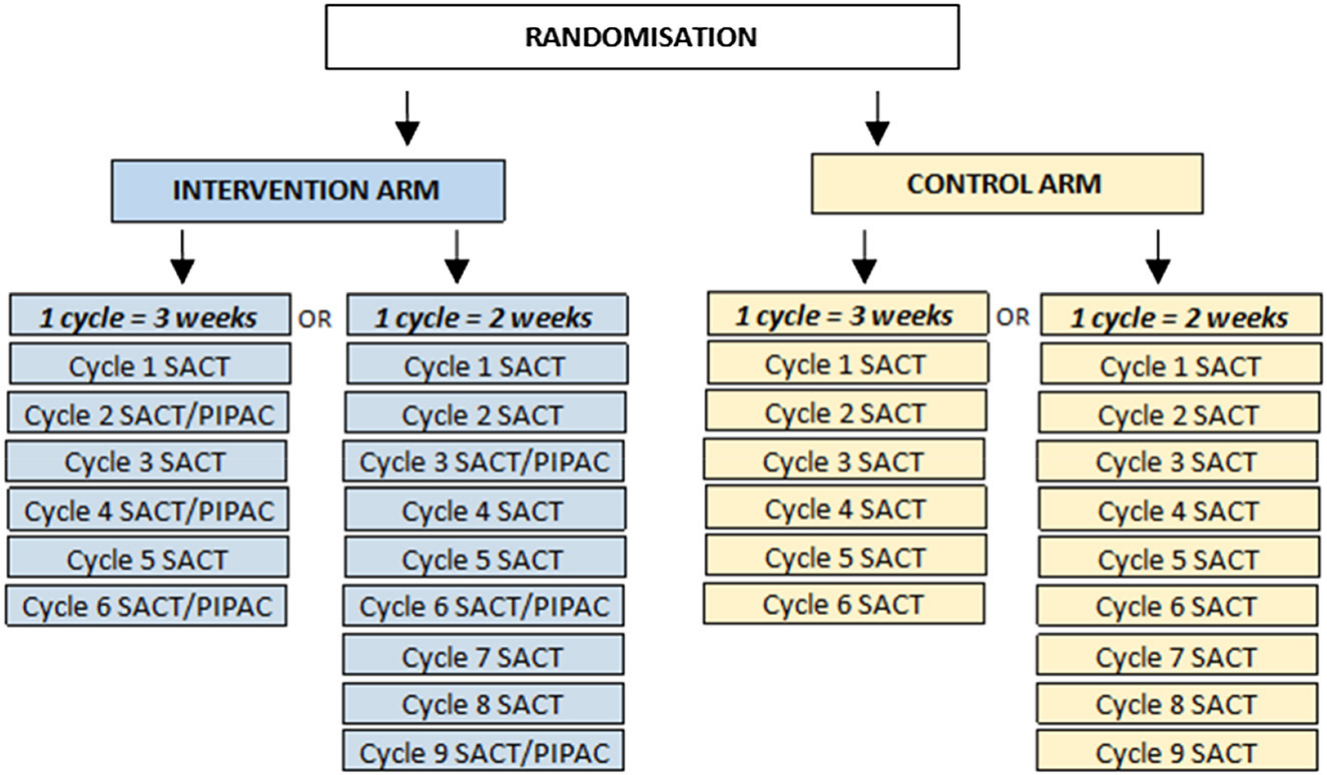
Colorectal group treatment schema.

Participants will receive up to three PIPAC treatments, 6 weeks apart with physician best choice SACT (see [Sec j_pp-2025-0023_s_005] for regimens) (Figure 2).

The PIPAC administered will be oxaliplatin 120 mg/m^2^, reduced to 90 mg/m^2^ in patients with frailty, intolerance or neuropathy. Mitomycin C 7.5 mg/m^2^ can be used as an alternative agent in patients with known, or who develop, hypersensitivity to oxaliplatin.

#### Ovarian intervention arm

**Figure 3:**
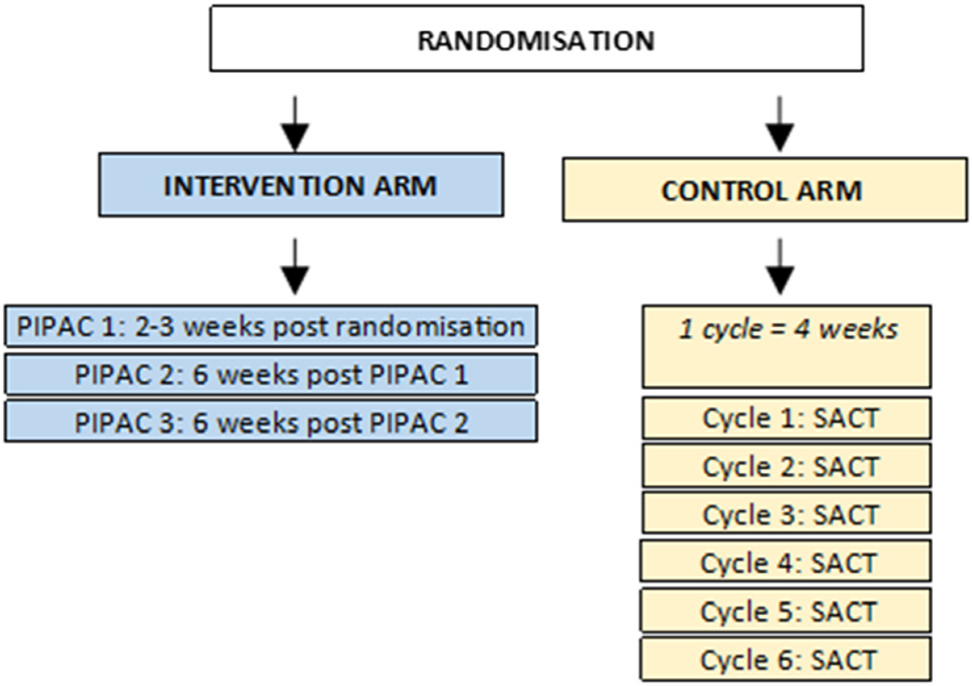
Ovarian group treatment schema.

Participants will receive up to three PIPAC treatments 6 weeks apart without SACT (Figure 3). The PIPAC administered will be a combination of cisplatin 10.5 mg/m^2^ and doxorubicin 2.1 mg/m^2^. Participants who show extraperitoneal progression and have not yet completed the treatment period may have SACT added in, at the discretion of their PI. If chemotherapy is added to the intervention arm, it should be completed 7 days prior to the PIPAC procedure (see supplementary material A – Ovarian group treatment schema and dosing schedule).

#### Gastric intervention arm

**Figure 4:**
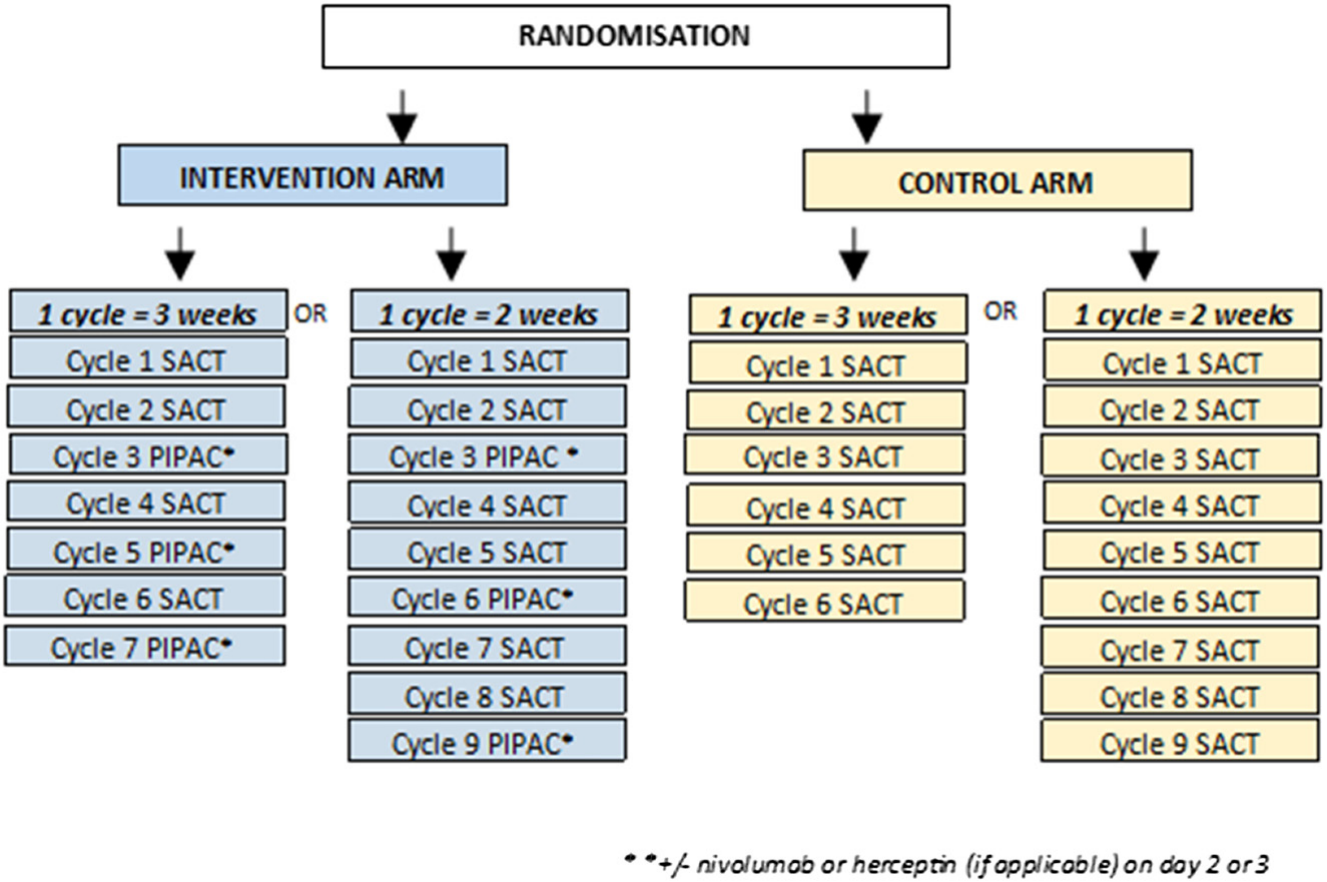
Gastric group treatment schema.

Participants will receive up to three PIPAC, treatments, 6 weeks apart with physician best choice SACT (see supplementary material A for regimens) (Figure 4). The PIPAC administered will be a combination of cisplatin 10.5 mg/m^2^ and doxorubicin 2.1 mg/m^2^.

### Pressurised intraperitoneal chemotherapy (PIPAC)

Surgeons providing PIPAC will have attended the International Society for the Study of Pleura and Peritoneum (ISSPP) PIPAC training course. PIPAC will be administered in accordance with the PICCOS PIPAC manual adhering to the published consensus statement for treatment protocols in PIPAC [39]. In brief, the procedure involves achieving a 12 mmHg pneumoperitoneum via an initial balloon safety trocar and insertion of additional balloon safety trocars into the abdominal wall. Ascites volume is suctioned and documented. The PCI score is recorded. Four quadrant peritoneal biopsies can be taken for pathology assessment (but are not required as part of the trial). The PIPAC nebuliser is connected to a high-pressure injector and inserted into the abdomen. All tubing is enclosed within laparoscopic camera plastic sheaths to ensure that there is no leak, and zero-flow of carbon dioxide is maintained throughout the procedure to ensure that the pneumoperitoneum is secure. Outlet tubing is connected to the trocar to allow secure exsufflation upon completion via a closed aerosol waste system (CAWS). The high-pressure injector parameters (flow rate) are set in line with the guidelines provided for the nebuliser being used with a maximum upstream pressure of 200 psi. The PICCOS modified ISSPP safety checklist is systematically double-checked before administration of PIPAC. The type, dose and preparation of chemotherapy will depend on the participants’ treatment group. Therapeutic pneumoperitoneum is maintained for 30 min then, the chemotherapy aerosol is exsufflated via CAWS. Finally, the trocars are extracted, and laparoscopy ended.

### Outcome measures

#### Primary outcome measure

Peritoneal progression free survival (pPFS) is defined as the interval between the date of randomisation and the first radiologically documented progression of peritoneal disease or death from any cause, patients with no events will be censored at the date of their last evaluable CT scan. Computed tomography (CT) scans performed according to disease group schedules will be used to monitor disease response. All CT images will undergo central review for RECIST assessment using a modified RECIST V1.1 by an expert radiologist review panel for the primary endpoint.

#### Secondary outcome measures

– QoL (EORTC QLQ C30 [40] plus three additional peritoneal disease specific EORTC items – fear of recurrence, abdominal pain and satisfaction with the healthcare team).
– Safety and surgical complication rates will be assessed for the duration of treatment and follow up and include:
  − Number of patients reporting toxicities, and worst grade reported, according to NCI CTCAE V5.0.
  − Total Number of episodes of neutropenic sepsis.
  − Number of patients reporting post operative complications through Clavien Dindo classification [41] within 30 days of each PIPAC, and worst grade reported for each type of complication.
  − Incidence of radiologically proven bowel obstruction.
– Proportion of intervention arm patients completing three PIPAC procedures.
– Number of conversions to operable disease in stomach or colorectal cancer.
– OS defined as days from randomisation to death from any cause. Patients still alive at the end of the trial will be censored at the date last seen.
– PFS defined as the number of days from date of randomisation to the date of progression (anywhere in the patient) or death from any cause.
– Extraperitoneal Progression Free Survival (ePFS), defined as the number of days from date of randomisation to the date of progression (outside of the peritoneum), death from any cause.
– Episodes of therapeutic ascitic drainages (in ovarian cancer) over the duration of treatment and follow-up.
– Peritoneal specific ORR observed at any time during treatment and follow-up.
– Peritoneal specific DCR defined as the proportion of patients with complete response, partial response or stable disease maintained at end of treatment scan (i.e. 3rd scan).

#### Tertiary/exploratory outcome measures

– The proportion of eligible patients who consent to randomisation out of those invited to take part.
– CEA (in colorectal cancer) and CA125 (in ovarian cancer) response from baseline to the end of follow up.
– PCI scoring at laparoscopy.
– Details of anti-cancer treatment participants have had after trial treatment has ended, until the end of follow-up.

### Participant timeline

The duration of the treatment period varies according to disease group and arm:

– Colorectal group: Intervention arm = 18 weeks, Control arm = 18 weeks.
– Ovarian group: Intervention arm = 14–15 weeks, Control arm = 24 weeks.
– Gastric group: Intervention arm = 18 or 21 weeks, Control arm = 18 weeks.

At end of treatment, participants will have a telephone call and CT scans every 2 months in the first year and then three monthly thereafter until the end of follow-up (which is 6 months after the last recruited patient is randomised). All participants will be followed up to the point of peritoneal disease progression as per RECIST v1.1 or until the end of follow-up, whichever comes first.

### Sample size

We aim to recruit 78 colorectal, 66 ovarian and 72 gastric patients. A randomised phase II screening design has been used to detect whether PIPAC shows enough activity to warrant further assessment in confirmatory phase III trials [42]. The 1:1 randomisation allows PIPAC (with or without SACT) to be compared to a concurrent control group who will receive SACT. Each group is powered to detect a Hazard Ratio (HR) of 0.55, a large enough effect size at this phase II stage to warrant further investigation. Using a high power of 90 % means that we have a high chance of detecting a true improvement in PFS, but since confirmatory trials would be required, a 20 % probability of a false positive is acceptable in this setting. A one-sided test is also employed because we only need to demonstrate an improvement in our primary outcome at this stage. The artmenu package in Stata 17 was used to calculate the sample size.

#### Colorectal

In an individual patient data meta-analysis of 1,375 colorectal patients with PM, PFS was estimated to be 6 months with SOC [6]. There are no randomised data on the effect of PIPAC on PFS or OS in colorectal cancer patients. To demonstrate that PIPAC has enough activity in this group to warrant further phase III investigation, we need to show improvement in median PFS to 11 months (HR 0.55, 20 % one-sided significance, 90 % power). This requires 51 PFS events and 66 patients recruited over 2.5 years, with an additional 6 month follow-up at the end of recruitment. This is inflated to 78 to allow for early dropouts due to being unfit for PIPAC.

#### Ovarian

Median PFS is estimated at 3.4 months with SOC [8]. There are no randomised data on the expected PFS in the PIPAC group. To demonstrate PIPAC has activity in this group we need to show an improvement in PFS to 6 months (HR 0.55 20 % one-sided significance, 90 % power). This requires 52 events, and 58 patients recruited over 2.5 years, with an additional 6 month follow-up at the end of recruitment. This is inflated to 66 to allow for early dropouts due to being unfit for PIPAC.

#### Gastric

PFS is estimated at 5 months in the SOC arm, which is close to that assumed by the French PIPAC phase II trial in gastric cancer [15], 16]. To demonstrate PIPAC has activity in this group we need to show an improvement in PFS to 9 months (HR 0.55, 20 % one-sided significance, 90 % power). This requires 52 events in 63 patients, recruited over 2.5 years, with an additional 6 month follow-up at the end of recruitment. This is inflated to 72 to allow for early dropouts due to being unfit for PIPAC.

### Randomisation and recruitment

Participants will be randomised via the web-based central PICCOS database in a 1:1 ratio to either SOC SACT or the PIPAC (with or without SACT) intervention arm. Allocation will be balanced between the two arms through a minimisation algorithm. Minimisation factors include PIPAC centre, to ensure there is no disparity in per patient resource costs between PIPAC sites, and additional prognostic factors to avoid bias. Within each disease group, the first patient will be allocated at random, and then minimisation with a 20 % random element will be used to allocate the next participant to treatment, thereby reducing the overall imbalance across all prognostic factors. The intended recruitment period is 30 months.

### Data management

The Sponsor will act as data controller. Cardiff University, Imperial College London, GI Cancer Imaging Ltd and individual participating sites will act as data processors. Data management procedures will be documented in a trial specific Data Management Plan (DMP) in line with the Data Protection Impact Assessment section of the study risk assessment.

Study data will be collected and managed using REDCap electronic data capture tools hosted at Cardiff University. Paper case report forms (CRFs) will be used as backup should REDCap be inaccessible. Completed paper QoL questionnaires will be entered onto REDCap by site staff. Participating sites will log patient screening on site-specific electronic screening logs and send a redacted version via secure electronic transfer to the CTR for central monitoring purposes.

## Statistical analysis

### Statistical analysis plan

Patients who have not progressed in the peritoneum or died at the time of analysis will be censored at the date if the CT scan from the latest evaluable RECIST assessment. Patients who progress or die after missing at least two RECIST assessments will be censored at the CT date of the last evaluable RECIST assessment. Patients with no RECIST assessments evaluable for peritoneal disease will be censored at day 1 unless they die within two RECIST visits of baseline (colorectal = 21 weeks, ovarian = 20 weeks, gastric = 22 week, in which case death will count as a PFS event). pPFS will be estimated using the Kaplan–Meier method and will be described in terms of median pPFS per arm. The log-rank test, stratified by prognostic factors, will be used to compare PFS distributions in the two trial arms. A one-sided p-value will be calculated, if this is less than 0.2 and the outcome favours the PICCOS treatment group, sufficient statistical evidence of activity will be confirmed in the PIPAC arm to warrant further investigation. We will use multivariable Cox regression to estimate the adjusted HR and one-sided 80 % upper confidence interval, adjusted for key prognostic factors such as PCI (if the proportional hazards assumption is not violated). If there is evidence of non-proportional hazards, then we will calculate the restricted mean survival times in each group. Cox regression will be used to estimate the HR and associated 95 % confidence intervals for the secondary time-to-event endpoints of overall PFS and OS, but no formal log-rank tests will be performed for these. Overall PFS will consider any evidence of disease progression from both peritoneal and extra-peritoneal lesions. The peritoneal specific ORR and the peritoneal specific DCR will be calculated for each group with 95 % confidence intervals.

The mean difference in global health scores between groups and functioning scales will be summarised at the end of treatment QoL timepoint. The median global health scores from the QLQ-C30 will be calculated with the inter-quartile range (IQR) at each QoL time point and summarised graphically with boxplots over time for each group. Single symptom items of pain, fatigue, fear of recurrence, abdominal pain and satisfaction with the medical team will be summarised as above.

There are no planned interim analyses or stopping rules.

### Monitoring

PICCOS is a Type B investigational medicinal product (IMP) trial (slightly higher than the risk of standard medical care), thus medium monitoring levels will be employed following a risk-adapted approach.

Central monitoring will be conducted via routine data queries and quality control checks of CRFs and site participant screening logs. This will focus on accrual, consent, withdrawal, data integrity and data protection.

Ad-hoc triggered site monitoring visits will be conducted if required to address site-related good clinical practice (GCP) or contractual non-compliance. Non-compliance identified centrally or at site will be reported to Research Ethics Committee (REC), Medicines and Healthcare products Regulatory Agency (MHRA), the Sponsor and participating sites as applicable following CTR standard policies and procedures. The study is subject to inspection by the REC and MHRA as the regulatory bodies, and inspection and audit by CVUHB as Sponsor.

The trial has an independent Data Monitoring Committee (including a statistician, a surgeon with PIPAC experience and specialists in each of the three disease groups). A Trial Management Group (TMG) composed of CTR members, surgical and oncology leads and patient representatives for each disease group and research fellows meet every three months. The TMG will monitor progress of the trial including recruitment and retention rates, screening logs, safety reporting and other items that may vary depending on the timepoint of the trial. The Trial Steering Committee (TSC) (including two surgeons with experience of PIPAC) will review key elements of trial progress including recruitment, IDMC recommendations and overall management of the trial. It will meet at least once per annum.

## Conclusions

As the first phase II RCT investigating the efficacy and impact on QoL of PIPAC in the treatment of PM in these three disease groups, PICCOS aims to provide much needed, high-quality evidence on the efficacy of this novel method of chemotherapy delivery in treating PM thereby guiding clinical practice and further research. Patients with colorectal, ovarian and gastric cancer eligible for this study have a poor prognosis. We hypothesise that PIPAC will improve peritoneal disease and preserve QoL in these subsets of patients.

## Supplementary Material

Supplementary Material
