# Supplementary material for: PICCOS: study protocol for pressurised intraperitoneal aerosolised chemotherapy (PIPAC) in the management of cancers of the colon, ovary and stomach: a randomised controlled, phase II trial of efficacy in peritoneal metastases
Source: Pleura Peritoneum. 2026 May 1;11(2):55–65. doi: 10.1515/pp-2025-0023 (PMC13340826; doi:10.1515/pp-2025-0023)
Supplement: Supplementary file 1 — Supplementary Material [file j_pp-2025-0023_suppl_001.docx]

**Supplementary Material**

**Colorectal group SACT options**

| **Option No,** | **SACT regime** | **No SACT is to be given in PIPAC treatment cycles except:** |
| --- | --- | --- |
| **1** | **FOLFOX**  - *2 weekly cycles* | - 5FU infusion continuous infusion – to be administered with pump disconnection 24 hours pre PIPAC or pump attachment 48 hours after PIPAC. |
| **2** | **FOLFOX + cetuximab**  - *2 weekly cycles* | - 5FU infusion continuous infusion - to be administered with pump disconnection 24 hours pre PIPAC or pump attachment 48 hours after PIPAC. - Cetuximab |
| **3** | **FOLFOX + Panitumumab**  - *2 weekly cycles* | - 5FU infusion continuous infusion – to be administered with pump disconnection 24 hours pre PIPAC or pump attachment 48 hours after PIPAC. - Panitumumab |
| **4** | **FOLFIRI**  *- 2 weekly cycles* | - 5FU infusion continuous infusion - to be administered with pump disconnection 24 hours pre PIPAC or pump attachment 48 hours after PIPAC. |
| **5** | **FOLFIRI + Cetuximab**  - 2 weekly cycles | - 5FU infusion continuous infusion – to be administered with pump disconnection 24 hours pre PIPAC or pump attachment 48 hours after PIPAC. - Cetuximab |
| **6** | **FOLFIRI + Panitumumab**   - 2 weekly cycles | - 5FU infusion continuous infusion – to be administered with pump disconnection 24 hours pre PIPAC or pump attachment 48 hours after PIPAC. - Panitumumab |
| **7** | **CAPOX**  - 3 weekly cycles | - Capecitabine 1000mg/m^2^ bd for 14 days (days 1-14 of the cycle) PO. *Capecitabine to be omitted for 24 hours prior to the PIPAC procedure and for 48 hours afterwards.* |
| *FOLFOX = oxaliplatin (IV) + 5FU (IV bolus) + 5FU (IV infusion) + s*odium folinate (IV) or calcium folinate (IV)*, FOLFIRI = irinotecan (IV) + 5FU (IV bolus) + 5FU (IV infusion) + s*odium folinate (IV) or calcium folinate (IV)*,, CAPOX = oxaliplatin (IV) + capecitabine (PO)* | | |

**Ovarian group SACT options**

| **Option no.** | **SACT regime** |
| --- | --- |
| **1** | Paclitaxel   - 4 weekly cycles |
| **2** | Liposomal doxorubicin   - 4 weekly cycles |

Gastric group SACT options

| **Option No,** | **SACT regime** | **No SACT is to be given in PIPAC treatment cycles except:** |
| --- | --- | --- |
| **1** | **FOLFOX**   - 2 weekly cycles |  |
| **2** | **CAPOX**   - 3 weekly cycles |  |
| **3** | **CX** - 3 weekly cycles |  |
| **4** | **CXtraz***   - 3 weekly cycles | Trastuzumab to be continued throughout PIPAC cycles, given day 3 post PIPAC (as applicable) |
| **5** | **CAPOXtraz***   - 3 weekly cycles |  |
| **6** | **FOLFOXtraz***   - 2 weekly cycles |  |
| **7** | **CAPOXniv****   - 3 weekly cycles | Nivolumab to be continued throughout PIPAC cycles, given day 3 post PIPAC (as applicable) |
| **8** | **FOLFOXniv****   - 2 weekly cycles |  |
| *FOLFOX = oxaliplatin (IV) + 5FU (IV bolus) + 5FU (IV infusion) + s*odium folinate (IV) or calcium folinate (IV)*, CAPOX = oxaliplatin (IV) + capecitabine (PO)*  *CX = cisplatin (IV) + capecitabine (PO)* | | |

*Trastuzumab to be used as per current NICE recommendation. Maintenance trastuzumab may be continued upon completion of planned trial treatment (if clinically indicated)

** Nivolumab to be used as per current NICE recommendation. Maintenance nivolumab may be continued upon completion of planned trial treatment (if clinically indicated)

*(Trastuzumab /nivolumab maintenance should continue after completion of trial schema (if clinically indicated) as per SOC.)*
